# Discovery of new methylation markers to improve screening for cervical intraepithelial neoplasia grade 2/3

**DOI:** 10.1186/s13148-016-0196-3

**Published:** 2016-03-09

**Authors:** A. Boers, R. Wang, R. W. van Leeuwen, H. G. Klip, G. H. de Bock, H. Hollema, W. van Criekinge, T. de Meyer, S. Denil, A. G J. van der Zee, E. Schuuring, G. B. A. Wisman

**Affiliations:** Department of Gynecologic Oncology, internal postal code DA13, Cancer Reserch Center Groningen, University of Groningen, University Medical Center Groningen, PO box 30.001, 9700 RB Groningen, The Netherlands; Department of Epidemiology, Cancer Reserch Center Groningen, University of Groningen, University Medical Center Groningen, Groningen, The Netherlands; Department of Pathology, Cancer Reserch Center Groningen, University of Groningen, University Medical Center Groningen, Groningen, The Netherlands; Department of Mathematical Modeling, Statistics and Bioinformatics, Ghent University, Ghent, Belgium

**Keywords:** Cervical cancer screening, Cervical precancerous lesions, Human papillomavirus (HPV), Cervical scraping, MethylCap-seq, DNA methylation, Quantitative methylation-specific PCR (QMSP)

## Abstract

**Background:**

Assessment of DNA promoter methylation markers in cervical scrapings for the detection of cervical intraepithelial neoplasia (CIN) and cervical cancer is feasible, but finding methylation markers with both high sensitivity as well as high specificity remains a challenge. In this study, we aimed to identify new methylation markers for the detection of high-grade CIN (CIN2/3 or worse, CIN2+) by using innovative genome-wide methylation analysis (MethylCap-seq). We focused on diagnostic performance of methylation markers with high sensitivity and high specificity considering any methylation level as positive.

**Results:**

MethylCap-seq of normal cervices and CIN2/3 revealed 176 differentially methylated regions (DMRs) comprising 164 genes. After verification and validation of the 15 best discriminating genes with methylation-specific PCR (MSP), 9 genes showed significant differential methylation in an independent cohort of normal cervices versus CIN2/3 lesions (*p* < 0.05). For further diagnostic evaluation, these 9 markers were tested with quantitative MSP (QMSP) in cervical scrapings from 2 cohorts: (1) cervical carcinoma versus healthy controls and (2) patients referred from population-based screening with an abnormal Pap smear in whom also HPV status was determined. Methylation levels of 8/9 genes were significantly higher in carcinoma compared to normal scrapings. For all 8 genes, methylation levels increased with the severity of the underlying histological lesion in scrapings from patients referred with an abnormal Pap smear. In addition, the diagnostic performance was investigated, using these 8 new genes and 4 genes (previously identified by our group: *C13ORF18*, *JAM3*, *EPB41L3*, and *TERT*). In a triage setting (after a positive Pap smear), sensitivity for CIN2+ of the best combination of genes (*C13ORF18*/*JAM3*/*ANKRD18CP)* (74 %) was comparable to hrHPV testing (79 %), while specificity was significantly higher (76 % versus 42 %, *p* ≤ 0.05). In addition, in hrHPV-positive scrapings, sensitivity and specificity for CIN2+ of this best-performing combination was comparable to the population referred with abnormal Pap smear.

**Conclusions:**

We identified new CIN2/3-specific methylation markers using genome-wide DNA methylation analysis. The diagnostic performance of our new methylation panel shows higher specificity, which should result in prevention of unnecessary colposcopies for women referred with abnormal cytology. In addition, these newly found markers might be applied as a triage test in hrHPV-positive women from population-based screening. The next step before implementation in primary screening programs will be validation in population-based cohorts.

**Electronic supplementary material:**

The online version of this article (doi:10.1186/s13148-016-0196-3) contains supplementary material, which is available to authorized users.

## Background

Cervical cancer is characterized by a well-defined pre-malignant phase, cervical intraepithelial neoplasia (CIN). Identification of these high-grade CIN (HSIL) lesions by population-based screening programs and their subsequent treatment has led to a significant reduction of the incidence and mortality of cervical cancer [1, 2]. Cytology-based testing of cervical smears is the most widely used cervical cancer screening method but is not ideal, as the sensitivity for detection of CIN2 and higher (CIN2+) is only ~55 % [3–5, 51]. Cervical carcinogenesis is highly associated with high-risk human papillomavirus (hrHPV) [6]. Large randomized controlled trials have shown that the sensitivity of hrHPV testing is significantly higher than cytology testing [4, 7–10]. Based on these data, starting in 2016, the Netherlands is one of the first countries to implement hrHPV testing as primary cervical cancer population-based screening. However, the specificity of hrHPV testing, especially in a young screening population, is relatively low [3, 11–13], which may lead to unnecessary referrals to the gynecologist, anxiety in the false-positive women, and higher costs for the health-care system. Finally, in the near future, the prevalence of CIN and cervical cancer will probably decrease in countries that have introduced primary prevention with hrHPV vaccination. With this decrease in prevalence, the positive predictive value of the current screening tests will by definition decrease [14]. Therefore, other objective biomarkers with both high sensitivity as well as high specificity are needed as new screening tools for cervical cancer.

Different DNA methylation patterns in normal versus (pre)malignant lesions represent excellent targets for diagnostic approaches based on methylation-specific PCR (MSP). Promoter hypermethylation of tumor suppressor genes is an early event in cervical carcinogenesis and consequently hypermethylation analysis can be especially relevant for the early detection of cervical neoplasia [15–17]. Assessment of methylation markers in cervical scrapings for the detection of CIN and cervical cancer is feasible [17–23], but obtaining methylation markers with both high sensitivity as well as high specificity remains a challenge. Through the years, gradually more sophisticated approaches have been developed to identify new methylation markers on a genome-wide scale [24]. Amidst comparable studies from other groups, we have previously reported our experience with pharmacological unmasking of the promoter region combined with re-expression as analyzed by microarrays, high-throughput quantitative methylation-specific PCR (QMSP) on an OpenArray platform, and methyl-DNA immunoprecipitation followed by microarray analysis (MeDIP), resulting in the discovery and validation of the genes *C13ORF18*, *JAM3*, *EPB41L3*, and *TERT* [21, 22, 25]. The diagnostic performance of these genes showed sensitivities for detecting CIN2+ in a hrHPV-positive population between 43 and 71 % and specificities between 89 and 100 % [21]. However, most strategies (including ours) for discovering new methylation markers so far were based on the difference between cancer and normal tissue resulting in markers with high sensitivity for carcinoma, but with too low sensitivity for detecting CIN2/3 lesions [21, 22, 26, 27]. New and more specific innovative genome-wide methylation analysis of DNA from CIN2/3 lesions versus normal cervical tissue should result in (new) CIN2/3-sensitive and -specific methylation markers. MethylCap-seq uses methyl-binding domain (MBD) proteins to specifically enrich for methylated DNA, followed by sequencing [28, 29]. The higher affinity of the MBD complex for double-stranded CpG-methylated DNA results in a higher enrichment for methylated DNA sequences as compared to MeDIP analysis. Using next-generation sequencing, a unique methylome of each sample will be generated (MethylCap-seq). After identification of novel methylation markers for (pre)malignant cervical neoplasia through this approach, validation and diagnostic evaluation of these newly found markers can be performed.

The aim of the present study was to identify new methylation markers that can differentiate between normal cervices and CIN2/3 lesions using MethylCap-seq with high specificity and high sensitivity. To validate the diagnostic performance of the newly found methylation markers considering any methylation level as positive, cervical scrapings will be tested as triage test after either primary cytology or primary hrHPV analysis using QMSP. Because for most of the reported markers in literature a cut-off value was set above a certain methylation level in order to obtain high specificity, we will preferentially select differentially methylated markers without setting a cut-off allowing a test that should be more objective and easy to interpret.

## Results

### Identification of differential methylated genes by MethylCap-sequencing

**Fig. 1:**
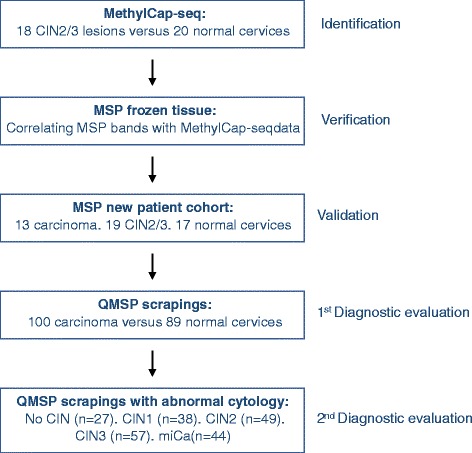
Flow scheme for the identification of new CIN2+ methylation markers

To identify new CIN2 or higher (CIN2+) specific methylation markers, we applied the strategy as shown in Fig. 1.

In short, genome-wide MethylCap-seq was used to compare the DNA methylation profiles of CIN2/3 dysplastic cervical cells with normal cervical cells to identify CIN2/3-specific differential methylated regions (DMRs). After applying our selection criteria (see “Methods” section for detailed description), 176 DMRs comprising 164 genes remained. The list of DMRs is shown in Additional file 1: Table S1, ranked on the sum of unmethylated normal samples and methylated CIN2/3 samples. The range of reads obtained for all these 176 DMRs of all samples considered methylation positive was from 3–17 reads with a median of 4 reads (data not shown).

### Verification and validation of the top 15 differentially methylated genes

To verify the MethylCap-seq data, the top 15, out of the 163 identified genes, were selected. Methylation-specific PCR (MSP) primers were designed and could be optimized for 14 out of the 15 genes. Verification of the selected 14 genes showed for 11 genes a significant correlation between the MSP band intensity and the amount of reads from the MethylCap-seq data. One gene (*PCDH17*) showed high methylation levels in leukocytes and was excluded for further validation in order to prevent false-positive methylation signals in samples of healthy women. The remaining 10 genes passed verification and continued to the subsequent validation step. Table 1 shows an overview of which genes continued through the different stages of validation.

**Table 1:** Verification, validation, and diagnostic evaluation of the highest ranking top 15 genes

Rank	Gene	Optimized	Verification	Validation	1st diagnostic evaluation	2nd diagnostic evaluation
1	*ZSCAN1*	Yes	Yes	Yes	Yes	Yes
2	*PCDH17*	Yes	No^a^			
3	*ST6GALNAC5*	Yes	Yes	Yes	Yes	Yes
4	*CLIC6*	Yes	No			
5	*RP11-89 K21.1.1*	Yes	No			
6	*ANKRD18CP*	Yes	Yes	Yes	Yes	Yes
7	*PAX2* ^b^	Yes	Yes	Yes	No	
8	*CDH6*	Yes	Yes	Yes	Yes	Yes
9	*GFRA1*	Yes	Yes	Yes	Yes	Yes
10	*IRX1*	Yes	No			
11	*POU4F3*	Yes	Yes	No^a^		
12	*GATA4*	Yes	Yes	Yes	Yes	Yes
13	*MKX*	No				
14	*PAX2* ^b^	Yes	No			
15	*KCNIP4*	Yes	Yes	Yes	Yes	Yes
16	*LHX8*	Yes	Yes	Yes	Yes	Yes

^a^Excluded due to high methylation in leukocytes

^b^Same gene, different region

The second validation step was performed by MSP on DNA isolated from FFPE tissue of an independent, randomly selected new patient cohort that consisted of 13 cervical cancers, 19 HSIL lesions (8 CIN2, 8 CIN3, and 3 adCIS) and 17 normal cervices. Out of the 10 genes analyzed, 9 were not methylated in almost all normal samples, significant differential methylation between normal versus HSIL lesions and again little to no methylation in the leukocytes (*p* < 0.05) (Table 2). These 9 genes (*ZSCAN1*, *ST6GALNAC5*, *ANKRD18CP*, *PAX2*, *CDH6*, *GFRA1*, *GATA4*, *KCNIP4*, and *LHX8*) were selected for further diagnostic evaluation in cervical scrapings (Table 2).

**Table 2:** Methylation positivity in an external cohort of FFPE samples to validate results of high methylation in CIN2+ lesions and no methylation in normal cervices of the newly found methylation markers

Gene	Normal	CIN2	CIN3	adCIS	Carcinoma
*ZSCAN1*	4/16	8/8	7/8	3/3	12/13
*ST6GALNAC5*	0/16	1/6	4/8	2/3	9/12
*ANKRD18CP*	0/16	1/8	1/7	2/3	6/12
*PAX2*	1/14	6/8	7/8	3/3	5/13
*CDH6*	1/15	3/8	4/8	3/3	7/13
*GFRA1*	0/12	2/8	3/8	2/3	10/12
*POU4F3* ^a^	2/14	6/7	3/7	3/3	11/12
*GATA4*	0/17	3/8	2/7	3/3	10/13
*KCNIP4*	0/17	6/8	5/8	3/3	10/12
*LHX8*	1/16	3/8	4/8	3/3	7/13

^a^Excluded due to high methylation in leukocytes

### Diagnostic evaluation by QMSP for normal versus cancer scrapings

To evaluate the diagnostic value of the new methylation markers (Table 2), cervical scrapings from two cohorts of patients were used: (1) normal versus carcinoma scrapings and (2) scrapings from patients referred from population-based screening with an abnormal Pap smear (≥pap2).

**Fig. 2:**
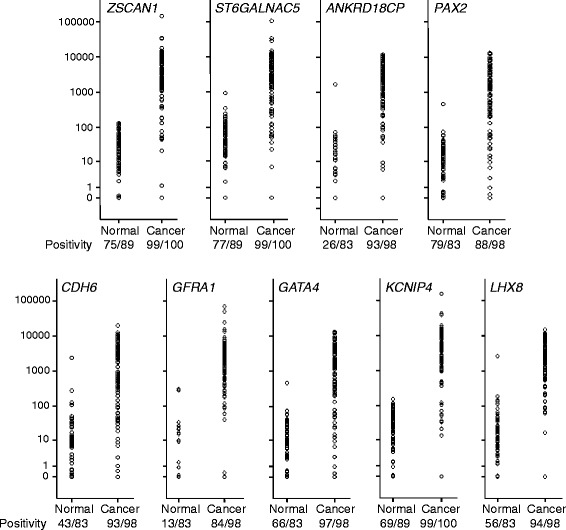
Methylation ratio of nine genes analyzed by QMSP in scrapings from normal (Nl) and cancer (Ca) patients. Methylation levels are significantly higher in the cancer scrapings (all genes, except *PAX2*, *p* < 0.001)

**Fig. 3:**
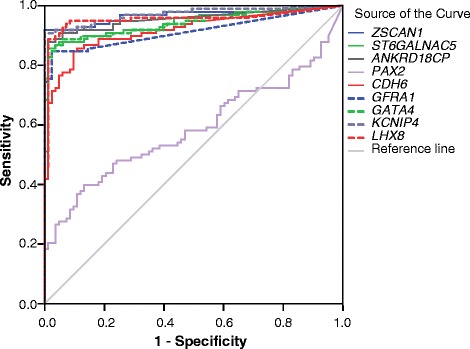
ROC analysis of methylation ratio per gene

In cohort 1, cervical scrapings of 100 randomly selected cervical carcinoma patients and of 89 patients with histologically confirmed normal cervices were used. QMSP analysis showed that the relative levels of DNA methylation were higher in the carcinoma scrapings compared to the normal scrapings for 8 out of the 9 selected genes (*p* < 0.001) (Fig. 2). The area under the curve (AUC) for methylation ratio in cervical carcinoma revealed for 8 genes an AUC > 0.91 (Fig. 3). Because of an AUC of 0.59, *PAX2* was excluded from further analysis.

### Diagnostic evaluation by QMSP for normal/LSIL versus HSIL scrapings

**Fig. 4:**
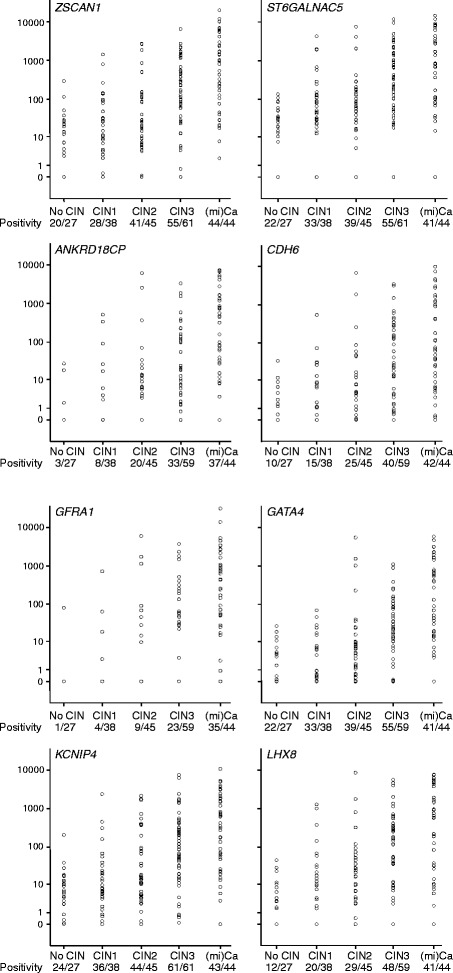
Methylation ratio of the eight genes analyzed by QMSP in scrapings from patients referred with an abnormal smear having a normal cervix (No CIN), CIN1, CIN2, CIN3, or (mi)Ca. Methylation levels increase significantly with the severity of the underlying lesion (all *p* < 0.001)

In cohort 2, scrapings of 215 consecutive patients referred from population-based screening with an abnormal Pap smear were used. The 8 genes that showed differential methylation in the normal versus the cancer scrapings were subsequently tested in this cohort. Methylation levels and frequencies for all 8 genes analyzed (*ZSCAN1*, *ST6GALNAC5*, *ANKRD18CP*, *CDH6*, *GFRA1*, *GATA4*, *KCNIP4*, and *LHX8*) increased with the severity of the underlying histological lesion (*p* < 0.001) (Fig. 4 and Table 3).

**Table 3:** Cytology according to the Papanicolaou system (Bethesda system) per histological subgroup. Methylation and HPV positivity of the 8 new methylation markers and 4 known markers tested with QMSP in cervical scrapings from patients with CIN0, CIN1, CIN2, CIN3, and (mi)Ca (*n* = 215)

Cytology	CIN0	CIN 1	CIN2	CIN3	miCa
Pap2 (ASCUS)	9/27 (33 %)	9/38 (24 %)	2/45 (4 %)	0	0
Pap3A (LSIL)	18/27 (66 %)	27/38 (71 %)	36/45 (80 %)	18/61 (30 %)	5/44 (11 %)
Pap3B (HSIL)	0	2/38 (5 %)	6/45 (13 %)	33/61 (54 %)	27/44 (61 %)
Pap4 (HSIL)	0	0	1/45 (2 %)	10/61 (16 %)	8/44 (18 %)
Pap5 (miCa)	0	0	0	0	3/44 (7 %)
Unknown	0	0	0	0	1/44 (2 %)
New genes					
*ZSCAN1*	20/27 (74 %)	28/38 (74 %)	41/45 (91 %)	55/61 (90 %)	44/44 (100 %)
*ST6GALNAC5*	22/27 (82 %)	33/38 (87 %)	39/45 (84 %)	56/61 (92 %)	41/44 (93 %)
*ANKRD18CP*	3/26 (12 %)	8/36 (22 %)	20/45 (44 %)	33/59 (56 %)	37/43 (86 %)
*CDH6*	10/26 (39 %)	15/36 (42 %)	25/45 (56 %)	40/59 (68 %)	42/43 (98 %)
*GFRA1*	1/26 (4 %)	4/36 (11 %)	9/45 (20 %)	23/59 (39 %)	35/43 (81 %)
*GATA4*	14/26 (54 %)	21/36 (58 %)	35/45 (78 %)	47/59 (80 %)	41/43 (95 %)
*KCNIP4*	24/27 (89 %)	36/38 (95 %)	44/45 (98 %)	61/61 (100 %)	43/44 (98 %)
*LHX8*	12/26 (46 %)	20/36 (56 %)	29/45 (64 %)	48/59 (81 %)	41/43 (95 %)
Known genes					
*C13ORF18*	2/27 (7 %)	1/38 (3 %)	9/45 (20 %)	24/61 (39 %)	27/44 (61 %)
*JAM3*	3/27 (11 %)	3/38 (8 %)	18/45 (40 %)	39/61 (64 %)	37/44 (84 %)
*EPB41L3*	2/27 (7 %)	12/38 (32 %)	18/45 (40 %)	44/61 (72 %)	41/44 (93 %)
*TERT*	13/27 (48 %)	22/38 (58 %)	29/45 (64 %)	51/61 (84 %)	43/44 (98 %)
HPV test					
hrHPV	12/26 (46 %)	24/36 (67 %)	36/45 (80 %)	49/59 (83 %)	31/43 (72 %)

Considering all detected methylation as positive to achieve higher sensitivity and/or specificity, genes *ZSCAN1*, *ST6GALNAC5*, *and KCNIP4* reached high sensitivity (≥90 %) for detection of CIN2+ lesions, while for *CDH6*, *GATA4* and *LHX8* sensitivity for CIN2+ was between 73 and 84 % (Table 4). For *ANKRD18CP* and *GFRA1*, sensitivity for CIN2+ was between 46 and 61 %, and these genes showed especially high specificity (82–92 %). In our analysis, we also included the 4 genes from our previously reported 4-gene panel (*C13ORF18*, *JAM3*, *EPB41L3*, and *TERT*) to compare sensitivity and specificity of these known genes with the newly identified methylation markers. The gene *C13ORF18* showed reproducible results as described previously [21] with high specificity (95 %) and relatively low sensitivity for CIN2+ of 40 %. *JAM3* and *EPB41L3* showed sensitivities for CIN2+ between 63 and 69 % and specificities between 79 and 91 %. The gene *TERT* was previously described with high specificity, but this result could not be reproduced since specificity was only 46 % in our present analysis, while sensitivity for CIN2+ lesions was 82 %.

**Table 4:** Sensitivity and specificity results for CIN2+ and CIN3+ in cervical scrapings from patients referred from population-based screening with an abnormal Pap smear (*n* = 215)

Gene	Sensitivity CIN2+	Specificity CIN2+	Sensitivity CIN3+	Specificity CIN3+
New genes				
*ZSCAN1*	93 %	26 %	94 %	19 %
*ST6GALNAC5*	90 %	15 %	92 %	16 %
*ANKRD18CP*	61 %	82 %	69 %	71 %
*CDH6*	73 %	60 %	80 %	53 %
*GFRA1*	46 %	92 %	57 %	87 %
*GATA4*	84 %	44 %	87 %	35 %
*KCNIP4*	99 %	8 %	99 %	6 %
*LHX8*	80 %	40 %	87 %	43 %
Known genes				
*C13ORF18*	40 %	95 %	49 %	89 %
*EPB41L3*	69 %	79 %	81 %	71 %
*JAM3*	63 %	91 %	72 %	78 %
*TERT*	82 %	46 %	90 %	42 %
HPV test				
hrHPV	79 %	42 %	78 %	33 %

### hrHPV status and triage testing

HrHPV testing was performed on the patient group referred with abnormal cytology at population-based screening. In 152/209 (73 %) samples, hrHPV could be detected; as for 6 out of 215 patients, insufficient material was available to perform HPV testing. Table 3 shows hrHPV status in relation to underlying histological diagnosis. HrHPV was present in 12/26 (46 %) patients without CIN lesion, 24/36 (67 %) CIN1 patients, 36/45 (80 %) CIN2 patients, 49/59 (83 %) CIN3 patients, and 31/43 (72 %) patients with micro-invasive cancer (miCa). The sensitivity of hrHPV testing for CIN2+ was 79 % with a specificity of 42 %. When comparing the sensitivity and specificity of hrHPV testing versus methylation marker testing, the genes *CDH6*, *GATA4*, and *LHX8* showed comparable sensitivity and specificity results to hrHPV testing with sensitivity for CIN2+ between 73 and 84 % and specificity between 40 and 60 % (Table 4).

Table 5 shows sensitivity and specificity for CIN2+ and CIN3+ in scrapings of hrHPV-positive women (*n* = 152), which were comparable to the results for the whole group, as shown in Table 4. The genes *ZSCAN1*, *ST6GALNAC5*, and *KCNIP4* again showed high sensitivity (≥92 %) for the detection of CIN2+, while for *CDH6*, *GATA4*, *EPB41L3*, *TERT*, and *ZSCAN16*, sensitivity for CIN2+ was between 72 and 85 %. For *ANKRD18CP*, *JAM3*, *C13ORF18*, and *GFRA1*, sensitivity for CIN2+ was between 43 and 68 %; however, these genes showed high specificity between 86 and 94 %. In the current Dutch population-based screening program, women with pap2/pap3a (ASCUS/LSIL) scrapings are retested after 6 months with triage testing by hrHPV. Therefore, we also show the results of triage testing by hrHPV and methylation markers in this group (Table 5). Triage testing by hrHPV shows a sensitivity for CIN2+ of 82 % with a specificity of 41 %; *GATA4*, *LHX8*, and *TERT* show comparable results.

**Table 5:** Sensitivity and specificity results for CIN2+ and CIN3+ in scrapings of hrHPV-positive women (*n* = 152). And in scraping of pap2/pap3a (ASCUS/LSIL) patients (*n* = 124)

	Sensitivity CIN2+	Specificity CIN2+	Sensitivity CIN3+	Specificity CIN3+
Only hrHPV-positive patients (*n* = 152)				
*ZSCAN1*	94 %	36 %	96 %	23 %
*ST6GALNAC5*	92 %	19 %	94 %	15 %
*ANKRD18CP*	65 %	86 %	74 %	71 %
*CDH6*	72 %	64 %	83 %	57 %
*GFRA1*	51 %	92 %	64 %	85 %
*GATA4*	85 %	47 %	88 %	33 %
*KCNIP4*	98 %	11 %	99 %	7 %
*LHX8*	81 %	53 %	91 %	47 %
*C13ORF18*	43 %	94 %	54 %	88 %
*EPB41L3*	72 %	78 %	85 %	68 %
*JAM3*	68 %	94 %	80 %	76 %
*TERT*	81 %	47 %	90 %	43 %
Only pap2/3a patients (*n* = 124)				
*ZSCAN1*	90 %	27 %	87 %	20 %
*ST6GALNAC5*	90 %	16 %	96 %	15 %
*ANKRD18CP*	38 %	82 %	41 %	75 %
*CDH6*	58 %	59 %	73 %	56 %
*GFRA1*	22 %	92 %	32 %	90 %
*GATA4*	78 %	44 %	77 %	35 %
*KCNIP4*	98 %	8 %	100 %	6 %
*LHX8*	70 %	49 %	86 %	46 %
*C13ORF18*	23 %	95 %	30 %	90 %
*EPB41L3*	51 %	78 %	74 %	72 %
*JAM3*	44 %	91 %	57 %	80 %
*TERT*	74 %	48 %	87 %	43 %
*hrHPV*	82 %	41 %	82 %	32 %

Different combinations of genes were analyzed to find the methylation marker panel with the highest combined sensitivity and specificity. A sample was considered positive if one of the genes in the combination tested was positive. By adding more than 3 genes in a combination, specificity of the methylation test decreased, with minimal increase in sensitivity. The combinations of genes with the highest combined sensitivity and specificity for CIN2+ were *JAM3/ANKRD18CP*, *C13ORF18/JAM3/ANKRD18CP*, and *JAM3/GFRA1/ANKRD18CP* with sensitivities of 72, 74, and 73 %, respectively, which are comparable to hrHPV testing (79 %). Specificity of the combinations were 79, 76, and 77 %, respectively, which are significantly higher than for hrHPV testing (42 %) (*p* ≤ 0.05). Table 6 shows that for all other combinations, sensitivities for detecting CIN2+ lesions are between 64 and 80 %, with a combined specificity between 58 and 88 %. For the detection of CIN3+ only, overall the sensitivity slightly increased (72–85 %), whereas the specificity decreased (48 and 72 %) (Table 6).

**Table 6:** Combinations of different methylation markers to create a panel of genes most suited as test in scrapings ranked on highest sensitivity (*n* = 215)

Gene combination	Sensitivity CIN2+	Specificity CIN2+	Sensitivity CIN3+	Specificity CIN3+
*JAM3/CDH6*	80 %	58 %	85 %	48 %
*ANKRD18CP/CDH6/EPB41L3*	80 %	55 %	87 %	48 %
*CDH6/EPB41L3*	78 %	57 %	85 %	50 %
*GFRA1/EPB41L3/CDH6*	78 %	57 %	85 %	50 %
*ANKRD18CP/CDH6*	77 %	57 %	83 %	49 %
*GFRA1/ANKRD18CP/CDH6*	77 %	57 %	83 %	49 %
*JAM3/EPB41L3/ANKRD18CP*	76 %	71 %	84 %	60 %
*C13ORF18/JAM3/ANKRD18CP* ^a^	74 %	76 %	80 %	62 %
*ANKRD18CP/EPB41L3*	74 %	74 %	83 %	64 %
*GFRA1/EPB41L3/ANKRD18CP*	74 %	74 %	84 %	64 %
*C13ORF18/CDH6*	74 %	58 %	80 %	51 %
*JAM3/GFRA1/ANKRD18CP* ^a^	73 %	77 %	80 %	64 %
*C13ORF18/JAM3/EPB41L3*	73 %	72 %	83 %	64 %
*GFRA1/CDH6*	73 %	60 %	80 %	53 %
*JAM3/ANKRD18CP* ^a^	72 %	79 %	79 %	65 %
*JAM3/EPB41L3*	72 %	75 %	83 %	66 %
*JAM3/EPB41L3/GFRA1*	72 %	76 %	83 %	66 %
*GFRA1/EPB41L3*	69 %	79 %	82 %	71 %
*C13ORF18/EPB41L3*	69 %	75 %	81 %	68 %
*C13ORF18/JAM3/GFRA1*	66 %	82 %	77 %	72 %
*JAM3/GFRA1*	65 %	86 %	76 %	75 %
*C13ORF18/ANKRD18CP*	65 %	79 %	72 %	67 %
*C13ORF18/JAM3*	64 %	88 %	73 %	76 %
*GFRA1/ANKRD18CP*	64 %	81 %	72 %	69 %

^a^These gene combinations showed the highest combined sensitivity and specificity

In hrHPV-positive scrapings, the sensitivities and specificities for CIN2+ of the 3 best-performing combinations (*JAM3/ANKRD18CP*, *C13ORF18/JAM3/ANKRD18CP*, and *JAM3/GFRA1/ANKRD18CP*) were comparable (sensitivity 76–77 %; specificity 81–83 %) (Table 7) to the population referred with an abnormal Pap smear. The sensitivity to detect CIN3+ of these 3 combinations again slightly increased to 85 %, while the specificity decreased 61–64 % (Table 7).

**Table 7:** Combinations of different methylation markers to create a panel of genes most suited as triage test in HPV-positive scrapings ranked on highest sensitivity (*n* = 152)

Gene combination	Sensitivity CIN2+	Specificity CIN2+	Sensitivity CIN3+	Specificity CIN3+
*JAM3/CDH6*	80 %	64 %	88 %	50 %
*ANKRD18CP/CDH6/EPB41L3*	79 %	61 %	89 %	51 %
*CDH6/EPB41L3*	77 %	61 %	88 %	53 %
*GFRA1/EPB41L3/CDH6*	78 %	61 %	88 %	53 %
*ANKRD18CP/CDH6*	77 %	61 %	85 %	51 %
*GFRA1/ANKRD18CP/CDH6*	77 %	61 %	85 %	51 %
*JAM3/EPB41L3/ANKRD18CP*	78 %	72 %	88 %	58 %
*C13ORF18/JAM3/ANKRD18CP* ^a^	77 %	81 %	85 %	61 %
*ANKRD18CP/EPB41L3*	75 %	75 %	86 %	63 %
*GFRA1/EPB41L3/ANKRD18CP*	75 %	75 %	86 %	63 %
*C13ORF18/CDH6*	73 %	61 %	83 %	54 %
*JAM3/GFRA1/ANKRD18CP* ^a^	76 %	81 %	85 %	63 %
*C13ORF18/JAM3/EPB41L3*	76 %	72 %	86 %	60 %
*GFRA1/CDH6*	72 %	64 %	83 %	57 %
*JAM3/ANKRD18CP* ^a^	76 %	83 %	85 %	64 %
*JAM3/EPB41L3*	75 %	75 %	86 %	63 %
*JAM3/EPB41L3/GFRA1*	75 %	75 %	86 %	63 %
*GFRA1/EPB41L3*	72 %	78 %	85 %	68 %
*C13ORF18/EPB41L3*	72 %	75 %	85 %	65 %
*C13ORF18/JAM3/GFRA1*	70 %	86 %	81 %	71 %
*JAM3/GFRA1*	69 %	89 %	81 %	74 %
*C13ORF18/ANKRD18CP*	68 %	83 %	76 %	67 %
*C13ORF18/JAM3*	69 %	92 %	80 %	74 %
*GFRA1/ANKRD18CP*	67 %	83 %	76 %	68 %

^a^These gene combinations showed the highest combined sensitivity and specificity

## Discussion

In this study, we report new CIN2/3-specific methylation markers identified by a genome-wide DNA methylation screening strategy comparing CIN2/3 and normal cervical cells. Diagnostic evaluation in cervical scrapings shows that for 8 newly identified genes, the relative level of methylation increases with the severity of the underlying histological lesion. Combining our newly identified genes with our previously reported panel (*C13ORF18*, *JAM3*, *EPB41L3*, and *TERT*) [21, 22] reveals that for the combinations *JAM3/ANKRD18CP*, *C13ORF18/JAM3/ANKRD18CP*, and *JAM3/GFRA1/ANKRD18CP*, sensitivities for CIN2+ were between 72 and 74 %, which was comparable to the sensitivity for CIN2+ of hrHPV testing (79 %). Specificities of these new gene panels were between 76 and 79 %, which was significantly higher (*p* ≤ 0.05) than the specificity for hrHPV testing (42 %) in a triage setting after a positive Pap smear test result in population-based screening. Furthermore, in hrHPV-positive scrapings, sensitivity and specificity for CIN2+ of these best-performing combinations were comparable to the population referred with an abnormal Pap smear.

Due to introduction of primary prevention of cervical cancer through prophylactic vaccination against hrHPV types 16 and 18, involved in 70 % of cervical cancers, the incidence of cervical neoplasia will decrease [14]. Current implementation of HPV vaccination programs in Europe will not have a real impact on the incidence of CIN2/3+ within the next 10–15 years. However, this decline in incidence will most probably impair the diagnostic performance of HPV testing and cytology triage testing even more, resulting in less efficient population-based screening programs [14]. There is therefore an urgent need to further improve current methodology for cervical cancer screening including new markers that are not associated with HPV.

Our strategy revealed 164 genes, of which the highest ranking 15 genes were validated in different steps. From the 164 identified genes, 10 were described previously in literature (*POU4F3*, *WT1*, *TBX3*, *SOX1*, *COL6A2*, *ALK*, *SOX17*, *PCDH10*, *CTNND2*, *APOBEC2*) as being more frequently methylated in CIN2/3 lesions and/or cervical cancer compared to normal cervices, indicating the validity of our approach. Of these 10 genes, *POU4F3* was further selected for validation in our approach, although we could not confirm the data already reported [52]. This might be due to another primer design as we used the DMR identified by MethylCap-seq. More CIN2+-specific markers are necessary since literature shows that methylation markers were often tested on CIN3 scrapings only and did not analyze scrapings of CIN2 patients [19, 20, 30].

In the current Dutch population-based screening program, women with pap2/pap3a (ASCUS/LSIL) scrapings are retested, after 6 months with triage testing by hrHPV. We showed that some single methylation markers had similar sensitivity and specificity for CIN2+ as hrHPV testing. However, combining methylation markers might improve the sensitivity but mostly resulting in a decrease of specificity.

Cervical cancer screening will change to primary hrHPV screening in the Netherlands in 2016. Because of the relatively low specificity of the hrHPV test, a triage test is necessary for hrHPV-positive women to prevent unnecessary referral to the gynecologist. Although triage testing with cytology is now mostly advocated, this test has some disadvantages because of its subjectivity and unreliability to test on material collected with self-sampling [31]. Therefore, we analyzed the performance of our methylation markers also in hrHPV-positive scrapings. The combination *JAM3/ANKRD18CP*, *C13ORF18/JAM3/ANKRD18CP*, and *JAM3/GFRA1/ANKRD18CP* showed in the hrHPV-positive scrapings sensitivities for CIN2+ between 76 and 77 % and specificities between 81 and 83 %. These results are better than for other reported triage strategies in literature, such as immunohistochemical staining with p16INK4a and/or KI67 that report sensitivities for detecting CIN2/3 77–87 % with a specificity of ~60 % [32–34] or for HPV 16/18 genotyping which reports sensitivity for CIN2/3 around 65 % with a specificity of 73 % [35]. Since our methylation panel was tested in a selected patient group that was referred from population-based screening with abnormal cytology, further validation in hrHPV-positive scrapings collected from a large cohort of women from population-based screening should be performed. These kinds of scrapings from real-life cohorts will become available in the Netherlands after 2016 when primary screening has changed to hrHPV testing.

The advantage of methylation marker analysis is that it is an objective test and can be performed on the same material used for hrHPV testing, which makes it also interesting for self-sampled material [36–38]. Different methylation markers already have been tested as a triage test in hrHPV-positive women [19, 21, 30, 39–42]. However, for most of these markers, a cut-off value was set above a certain methylation level in order to obtain high specificity. The advantage of our methylation panel is that no cut-off value is needed. If the PCR product is negative (i.e., no amplification of specific product), the samples are called negative and any ratio above zero is called positive. This unique feature of the selected genes allows an objective and easy to interpret test. However, although we focused on the methylation markers for which no cut-off was required, the other identified markers are still of interest for further investigation.

The gene *ANKRD18CP* (ankyrin repeat domain 18C, pseudogene) is located on chromosome 9 and its function is still unknown. Moreover, methylation of *ANKRD18CP* was not described before in any type of cancer. The gene *CDH6* (Cadherin 6) belongs to the family of cadherins. Cadherins are membrane glycoproteins that mediate homophilic cell–cell adhesion and play critical roles in cell differentiation and morphogenesis. Decreased expression of this gene may be associated with tumor growth and metastasis. It has recently been described as a new transforming growth factor-β (TGF-β) target in thyroid tumor patients [43]. The gene *GFRA1* (GDNF family receptor alpha 1) plays a key role in the control of neuron survival and differentiation. It has been described as differentially methylated between cancerous and non-cancerous tissue obtained from lung cancer patients based on DNA methylation profiles [44].

The strengths of our current study are (1) the genome-wide approach with MethylCap-seq for specific identification of differential methylation regions between normal cervices and CIN2/3 lesions, (2) the systematic verification and validation of the markers found using carefully revealed cohorts, and (3) the selection of the best-performing markers for diagnostic evaluation in cervical scrapings. The limitation of the current study was that the diagnostic evaluation of the markers could not be performed on women with HPV-positive scrapings with normal cytology, because referral for colposcopy with biopsies of this specific group is presently not allowed by law in the Netherlands. Importantly, it has been shown that approximately 25 % of the CIN2+ cases are hrHPV positive with a subsequent normal cytology [10, 42]. To study the effect of our results reported in this manuscript is subject of future investigations.

Population-based screening is in transition and methylation markers might be an important component in future screening settings. It is important to validate the most interesting markers described in literature in a population-based screening trial. Verification of the results by different groups is important to assure the reproducibility of the methylation analysis. The combination of genes with the highest possible sensitivity and specificity should be evaluated.

## Conclusions

In conclusion, we identified several new CIN2/3-specific methylation markers for detection of cervical neoplasia in cervical scrapings. These newly found markers might be applied as a triage test in hrHPV-positive women from population-based screening.

## Methods

### General strategy

To characterize the DNA methylome of CIN2/3 lesions and to identify new CIN2 or higher (CIN2+) methylation markers, we applied the following strategy (see Fig. 1): First, methylated DNA was enriched using MBD proteins with subsequent paired-end sequencing (MethylCap-seq) on DNA isolated from fresh-frozen macro-dissected epithelial tissue of 18 CIN2/3 lesions (6 CIN2 and 12 CIN3), 20 normal cervices, and two pools of leukocyte DNA of healthy volunteers. In order to identify differential methylated regions (DMRs), we retrieved the reads of promoter and exon regions. We selected methylation markers that showed clear-cut differences between the normal and CIN2/3 cervices, while also the leukocyte count had to be low, to prevent false-positive results. Markers were ranked on high specificity (no methylation in the normal cervices) and high sensitivity (methylation in CIN2/3 lesions). For the highest ranking top 15 genes, MSP primers were designed and methylation patterns were verified on the same DNA, which originally was used for MethylCap-seq. This first validation step enabled verification of MethylCap-seq data by correlating MSP band intensity with the number of reads from the MethylCap-seq. In the second validation step, high prevalence of methylation in the CIN2/3 lesions and no methylation in the normal cervices were analyzed by MSP analysis on DNA isolated from a completely independent cohort of patients (cervical cancer (*n* = 13), CIN2/3 lesions (*n* = 19), and normal cervices (*n* = 17)). DNA was isolated from macro-dissected formalin-fixed paraffin-embedded (FFPE) epithelial tissue.

Finally, diagnostic evaluation of the newly discovered methylation markers was performed by QMSP on cervical scrapings. First, we tested the methylation ratios of new biomarkers on a large series of randomly selected scrapings from cervical cancer patients (*n* = 100) and a similar age group of healthy controls (*n* = 89). Secondly, the potential of the new methylation markers as a diagnostic tool was evaluated in a large series of scrapings (*n* = 215) of randomly selected patients, referred with an abnormal Pap smear at population-based screening. Histology was used as the reference standard.

### Patient samples

All patients referred to the outpatient clinic of the University Medical Center Groningen (UMCG) with cervical cancer or an abnormal Pap smear at population-based screening are routinely asked to participate in our ongoing “methylation study” which has been approved by the institutional review board (IRB) of the UMCG. Cervical tissue, scrapings, and clinicopathologic data are prospectively collected and stored in our tissue bank. Within our methylation study tissue samples, scrapings and clinicopathologic data from normal cervices are also collected from patients who planned to undergo a hysterectomy for non-malignant reasons. All cervical tissue that was used for the normal control group was judged as histopathologically normal. Patients referred with cervical cancer are staged according to the FIGO criteria with pelvic examination and biopsies under general anesthesia. Cervical scrapings from both groups (cervical cancer staging and benign gynecologic surgery) were collected before surgery under general anesthesia. All patients referred with an abnormal Pap smear at population-based screening underwent an additional Pap smear prior to colposcopy specifically for this study. In this last cohort, women were only eligible when referred with an abnormal smear at population-based screening and not when they were referred to our hospital with complaints. At colposcopy, biopsies and/or large loop excision of the transformation zone (LLETZ) were performed. The tissue samples were scored by an experienced gynecologic pathologist, and the histological classification was used as the reference standard. If no interference with routine diagnostic evaluation was anticipated, specimens from the CIN lesions were retrieved and stored at −80 °C. Clinicopathologic data were retrieved from patient files and stored in our large anonymous password-protected institutional gynecologic oncology database. All patients gave written informed consent.

For the frozen tissue samples used in the MethylCap-seq analysis, the median age of the CIN2/3 patients was 35 years (interquartile range (IQR) 30–39) and for the patients with normal cervices 43 years (IQR 41–44). For the independent cohort of patients with FFPE samples, the median age of the CIN2/3 patients was 37 years (IQR 34–41), for the patients with normal cervices 43 years (IQR 40–44), and for the cervical cancer patients 49 years (IQR 42–54). For the cervical scrapings, the median age of cervical cancer patients was 50 years (IQR 39–64) and for the patients with normal cervices 47 years (IQR 43–53). The stages of cervical cancer patients were 1 (1 %) FIGO stage IA1, 31 (31 %) FIGO stage IB1, 18 (18 %) FIGO stage IB2, 21 (21 %) FIGO stage IIA, 17 (17 %) FIGO stage IIB, 1 (1 %) FIGO stage IIIA, 8 (8 %) FIGO stage IIIB, and 3 (3 %) FIGO stage IV. Histological classifications of the cervical cancer patients were 70 (70 %) squamous cell carcinoma (SCC), 21 (21 %) adenocarcinoma (AD), 3 (3 %) adenosquamous carcinoma (ASC), and 6 (6 %) undifferentiated carcinoma. The median age of the patients referred with an abnormal Pap smear was 37 years (IQR 32–43). The histological classifications of these patients were 27 without CIN, 38 CIN1, 45 CIN2, 61 CIN3, and 44 miCa (29 SCC, 12 AD, 3 ASC). The Pap smears were classified according to the Papanicolaou system. Table 3 shows, per histological subgroup, the Pap classification (and translation to Bethesda).

From all frozen tissue samples used for MethylCap-seq and the FFPE samples, 10-μm tissue sections were cut and macro-dissection was performed to enrich for epithelial cells. Before and after cutting, a hematoxylin and eosin slide was made to check the presence of epithelial cells. Cervical scrapings were collected in 5-ml ice-cold phosphate-buffered saline (PBS 6.4 mM NA_2_HPO_4_; 1.5 mM KH_2_PO_4_; 0.14 M NaCl; 2.7 mM KCl) and kept on ice until further processing. Of these 5-ml cell suspensions, 1 ml was used for cytomorphological assessment. The remaining 4 ml was centrifuged and the cell pellet was suspended in 1-ml TRAP wash buffer and divided in 4 fractions. Two fractions were stored as dry pellet at −80 °C for DNA isolation as described previously [21].

### DNA isolation

Tissue slides from FFPE tissue were deparaffinized using 100 % xylene followed by 100 % ethanol [17]. Genomic DNA from fresh-frozen macro-dissected samples and cervical scrapings was isolated by standard overnight 1 % SDS and proteinase K treatment, salt-chloroform extraction, and isopropanol precipitation as described previously [21]. DNA pellets were washed with 70 % ethanol and dissolved in 150 μl TE^−4^ (10 mM Tris/HCL; 0.1 mM EDTA, pH 8.0). Genomic DNA was amplified in a multiplex PCR according to the BIOMED-2 protocol, to check the DNA’s structural integrity [45]. For the MethylCap-seq samples, DNA quantity was measured using Quant-iT™ PicoGreen® dsDNA Assay Kit according to manufacturer’s protocol (Invitrogen, Carlsbad, CA, USA). For cervical scrapings, DNA concentrations and 260/280 ratios were measured using the Nanodrop ND-1000 Spectrophotometer (Thermo Scientific, Waltham, MA, USA). A 260/280 ratio of >1.8 was required for all samples.

### CpG-methylated island DNA capturing followed by next-generation sequencing (MethylCap-seq)

Methylated DNA fragments were captured with methyl-binding domains using the MethylCap kit according to manufacturer’s instructions (Diagenode, Liège, Belgium). The kit consists of the methyl-binding domain (MBD) of human MeCP2, as a C-terminal fusion with glutathione-S-transferase containing an N-terminal His6-tag. Before capturing, DNA samples (500 ng) were sheared to a size range of 300–1000 bps using a Bioruptor™ UCD-200 (Diagenode, Liège, Belgium) and fragments of ~300 bp were isolated. Leukocyte DNA of 4 healthy controls were included in 2 sets of 2 samples. Captured DNA was paired-end-sequenced on the Illumina Genome Analyzer II platform according to protocol (Illumina, San Diego, CA, USA). Results were mapped on the nucleotide sequence using Bowtie software [46], visualized using BioBix’ H2G2 browser (http://h2g2.ugent.be/biobix.html) and processed using the human reference genome (NCBI build 37). The paired-end fragments were unique and located within 400 bp of each other [47].

### MethylCap-sequencing analysis

For statistical analysis, reads of promoter (−2000 bp to +500 bp of transcription start site) and exon regions were retrieved. In order to identify differences between normal cervices and CIN2/3 lesions, we dichotomized the read data into methylation positive or negative. Samples were considered negative if a sample showed either 0 or 1 read. Samples were considered methylation positive if a sample showed ≥3 reads in order to be more confident that these regions were truly methylated. Subsequently, regions were ranked based on highest specificity and highest sensitivity for CIN2/3. The candidate markers should fulfill the following criteria: (1) low/negative reads in the leukocytes to prevent false-positive results. The region was excluded if both leukocyte samples showed >1 read or if 1 leukocyte sample showed >2 reads. (2) Markers should be unmethylated (0 or 1 read) in at least 75 % (15/20) of the normal cervix group. (3) Markers should be methylated (≥3 reads) in at least 28 % (5/18) of the CIN2/3 lesion group.

### Verification and validation of MethylCap-sequencing data by methylation specific PCR (MSP)

MSP primers were designed for the highest ranking top 15 genes (16 DMRs). Sodium bisulfite treatment of isolated genomic DNA (1 μg/sample) was performed according to the recommendations of the EZ DNA methylation kit (Zymo, BaseClear, Leiden, the Netherlands). MSP design and analysis was performed using sequences derived from the H2G2 browser. Each reaction was performed in 30-μl total reaction volume, containing: 600 nM of each MSP primer, 1.5 μl of bisulfite-treated DNA (approximately 15 ng), standard PCR components (Applied Biosystems), and 0.5 U AmpliTaq Gold DNA polymerase (Applied Biosystems). Condition of the MSP was 10 min hot-start at 95 °C; 95 °C for 60 s, 60 °C for 60 s, and 72 °C 60 s for a total of 40 cycles, with a final elongation step of 7 min at 72 °C. Leukocyte DNA from healthy women was used as negative control and in vitro methylated (by *SssI* enzyme) leukocyte DNA was used as positive control for each MSP.

### Quantitative methylation-specific PCR (QMSP)

QMSP was performed as described previously by our group with an internal (FAM-ZEN/IBFQ)-labeled hybridization probe for quantitative analyses [21]. Primer and probe sequences are available upon request. *β–actin* was used as a methylation independent internal reference gene. QMSP reactions were performed in 10 μl final volume, containing: 300 nM of forward and reverse primers, 250 nM of hybridization probe, 5 μl of 2* QuantiTect Probe PCR Master Mix (Qiagen, Hilden, Germany) and 2.5 μl bisulfite modified DNA (approximately 25 ng). Each sample was analyzed in triplicate by ABI PRISM® 7900HT Sequence Detection System (Applied Biosystems). Negative and positive controls were the same as used for MSP. Standard curve analysis was performed on each plate and by each primer-probe set on serial dilutions of in vitro methylated leukocyte DNA. A DNA sample was considered methylated if at least 2 out of the 3 wells were methylation positive with a Ct value below 50 and DNA input of at least 225 pg β-actin. The relative level of methylation of the region of interest was determined by the following calculation: the average quantity of the methylated region of interest divided by the average quantity of the reference β-actin gene and multiplied by 10,000 [48]. In our analysis, we also included 4 genes previously described by our group (*C13ORF18*, *JAM3*, *EPB41L3*, and *TERT*) to compare sensitivity and specificity of these known genes with the newly identified methylation markers. QMSP for these markers was performed as previously described [21].

### HPV testing

HrHPV testing was performed using general primer-mediated PCR (GP5+/6+) as reported previously [48]. For HPV typing as well as detection of the clinical relevant HPV infections, GP5+/6+-positive cases were tested by COBAS® 4800 HPV test. The COBAS HPV test individually detects HPV types 16 and 18, while at the same time identifying 12 additional hrHPV types [49]. The COBAS HPV test is routinely used in our iso-15189-certified laboratory of molecular pathology on scrapings from the national population-based screening program. For the COBAS® HPV testing in this study, the PCR-only workflow was used, since no liquid-based scrapings in Preservcyt® were available but only already isolated DNA. This workflow was first validated with DNA isolated from clinical samples that were tested previously in the diagnostic routine and this showed comparable results to the liquid-based samples.

### Statistical analysis

Statistical analysis was performed using SPSS software package (SPSS 20, Chicago, IL, USA).

Spearman’s rank correlation coefficient was used to compare the MethylCap-seq reads with the MSP band intensity. Categorical methylation data were analyzed using the Pearson *χ*^2^ test. Receiver operating characteristic (ROC) curves were generated and the area under the ROC curve (AUC) was used as a measure of test performance. The Mann–Whitney *U* test and Kruskall–Wallis test was used to determine differences in methylation ratio in 2 groups or more, respectively. The student *t* test was used to compare positive methylation and age. To compare sensitivity and specificity of the patient group referred with abnormal cytology by DNA methylation markers versus hrHPV, the extended McNemar test, described by Hawass, was executed [50]. *p* values lower than 0.05 were considered statistically significant.

## Additional file

Additional file 1: Table S1. Identified DMR’s with sensitivity and specificity ranked on the sum of both. (XLSX 21 kb)

## Abbreviations

ANKRD18CP: ankyrin repeat domain 18C, pseudogene
AUC: area under the ROC curve
CDH6: cadherin 6
CIN: cervical intraepithelial neoplasia
CIN2+: CIN2 and higher
DMR: differential methylated region
FFPE: formalin-fixed paraffin-embedded
GFRA1: GDNF family receptor alpha 1
hrHPV: high-risk human papillomavirus
HSIL: high-grade CIN
IQR: interquartile range
IRB: institutional review board
LLETZ: large loop excision of the transformation zone
MBD: methyl-binding domain
MeDIP: methyl-DNA immunoprecipitation
MSP: methylation-specific PCR
PBS: phosphate-buffered saline
QMP: quantitative MSP
ROC: receiver operating characteristic
UMCG: University Medical Center Groningen
